# A national physician survey on prescribing syringes as an HIV prevention measure

**DOI:** 10.1186/1747-597X-4-13

**Published:** 2009-06-08

**Authors:** GE Macalino, D Dhawan Sachdev, JD Rich, C Becker, LJ Tan, L Beletsky, S Burris

**Affiliations:** 1 Arthur Ashe Institute for Urban Health, 450 Clarkson Ave Box 1232, Brooklyn, New York 11203, USA; 2 Brown Medical School, Providence, Rhode Island 02912, USA; 3 The Miriam Hospital, 164 Summit Avenue, Providence, Rhode Island 02906, USA; 4 Brown University, Providence, Rhode Island 02912, USA; 5 American Medical Association, 515 N State Street, Chicago, Illinois 60610, USA; 6 Temple University School of Law, 1719 North Broad St Philadelphia, Pennsylvania 19122, USA; 7 Temple University School of Law, 1719 North Broad St Philadelphia, Pennsylvania 19122, USA

## Abstract

**Background:**

Access to sterile syringes is a proven means of reducing the transmission of human immunodeficiency virus (HIV), viral hepatitis, and bacterial infections among injection drug users. In many U.S. states and territories, drug paraphernalia and syringe prescription laws are barriers to syringe access for injection drug users (IDUs): pharmacists may be reluctant to sell syringes to suspected IDUs, and police may confiscate syringes or arrest IDUs who cannot demonstrate a "legitimate" medical need for the syringes they possess. These barriers can be addressed by physician prescription of syringes. This study evaluates physicians' willingness to prescribe syringes, using the theory of planned behavior to identify key behavioral influences.

**Methods:**

We mailed a survey to a representative sample of physicians from the American Medical Association physician database. Non-responding physicians were then called, faxed, or re-sent the survey, up to four times.

**Results:**

Twenty percent responded to the survey. Although less than 1 percent of respondents had ever prescribed syringes to a known injection drug user, more than 60% of respondents reported that they would be willing to do so. Physicians' willingness to prescribe syringes was best predicted by the belief that it was a feasible and effective intervention, but individual and peer attitudes were also significant.

**Conclusion:**

This was the first nationwide survey of the physician willingness to prescribe syringes to IDUs. While the majority of respondents were willing to consider syringe prescription in their clinical practices, multiple challenges need to be addressed in order to improve physician knowledge and attitudes toward IDUs.

## Background

**"**Because physicians are key gatekeepers to syringe access, efforts to encourage them to prescribe syringes to [Injection Drug Users] will help ensure that those who continue to inject can legally obtain sterile syringes. Involving physicians also illustrates the medical rationale for increasing IDUs' access – that improving access to sterile syringes has a legitimate medical purpose in preventing disease."[1]

Providing safe injection equipment to people who inject illicit drugs reduces the risk of transmission of the human immunodeficiency virus (HIV) and hepatitis C virus (HCV) and can encourage users to enter substance abuse treatment.[2] Data collected from the National Health and Nutrition Examination Survey from 1999–2001 found that of individuals reporting injection drug use in the past year, 47.1% purchased their last syringe at a pharmacy, 21.5% obtained the syringe privately from someone else, 8.7% bought their injection equipment on the street, 3.6% acquired it from a needle exchange, and the remaining obtained syringes at a shooting gallery or from a drug dealer.[3]

Laws and the attitudes of pharmacists and police officers continue to make it harder for IDUs to purchase or carry sterile syringes in many places. Only three states now require a prescription for all retail syringe purchases, but most states have drug paraphernalia laws or pharmacy regulations that may make pharmacists less willing to sell syringes to IDUs, and expose IDUs to police interference or arrest if they cannot demonstrate a legal basis for possessing syringes. [4-8]

Health care providers of IDUs are in a unique position to improve syringe access and increase sterile injection practices. [9,10] Physicians can legally prescribe syringes to IDU patients in most states. Even in locations where it is not legally required a medical prescription for obtaining a syringe may make it easier for IDUs to buy syringes in pharmacies and to avoid arrest or confiscation of syringes by the police. A pilot syringe prescription program among 327 IDUs in Rhode Island provided participants with basic clinical exams, medical care, hepatitis A and B immunizations, and links to substance abuse treatment. Notably, patients reported correct disposal in biohazard containers and decline in some drug-using behaviors. [11] Physicians who prescribe syringes can also effectively engage patients in conversations on drug use and drug treatment. Physician counsel can be a powerful influence on safe injection practices. [9-13]

Small-scale surveys conducted in Rhode Island and among addiction medicine specialists reported that a majority of physicians were receptive to the idea of prescribing syringes to IDUs. [13,14] The American Medical Association, Infectious Diseases Society of America, American Public Health Association, the Centers for Disease Control, and other professional and public health organizations have supported syringe prescription in conjunction with drug treatment education and counseling for IDUs. [1]

Much stands in the way, however, of the public health gains that would be made possible by physicians prescribing syringes. Barriers to syringe prescription could include the following: the stigma attached to drug use by physicians, lack of formal medical education about drug treatment options, lack of local availability of drug treatment and other interventions, time and financial pressures, professional traditions, fears that the patient base may disapprove of physicians who provide care to IDUs, and mutual mistrust between physicians and their drug using patients. [10,15,16]

This study was undertaken to assess physicians' willingness to prescribe syringes to IDU patients and to understand the barriers and facilitators to physician participation in such programs. We use the theory of planned behavior to identify factors influencing willingness to prescribe syringes, and offer recommendations for action to promote better health care for IDUs.

## Methods

### Study Sample

The study population was selected from all registered physician members of the American Medical Association (AMA), which includes physicians from all fifty states and Puerto Rico. We chose the study population from among physicians who either provided direct patient care or practiced in specialties that were likely to treat patients who could be injection drug users, including practitioners in the following categories: Primary care providers (Internal Medicine, Family Practice, General Practice, Gynecology); 2) Pediatric/adolescent medical providers; 3) Emergency room physicians; and 4) Specialists (Addiction Medicine, Addiction Psychiatry, Endocrinology, Diabetes and Metabolism, Infectious Disease, Obstetrics, Physical Medicine, Rehabilitation, and Pain Medicine). The AMA database has basic demographic information including gender, race, age, geographic location, and date of medical school graduation.

### Physician Data Collection

A four-page, 46-question survey was mailed to our study sample, including a cover letter describing the study and noting the sponsorship of the Centers for Disease Control and Prevention (CDC), the AMA, Brown Medical School, and Temple University Law School. Physicians were informed that their responses would be kept confidential, and consent was confirmed by the completion and submission of the survey. All mailings included a self-addressed return envelope with postage included. Up to four mailings were sent out to each participant, until a response was obtained. The first, second, and third mailings were sent by first class mail. The fourth mailing was sent by FedEx 2-day delivery. Concurrently, to increase response rate, we called physicians with available phone numbers. Physicians were asked if they had received a copy of the survey and, if not, we asked if we could fax them another copy. Up to 3 faxes were sent to each physician, up to three weeks after the initial mailing. The survey consisted of 17 multiple-choice questions and 28 agreement-items with a 5-point Likert scale response choice ("strongly agree" to "strongly disagree"). The multiple choice questions covered a range of subjects, including whether physicians treated IDUs in their practices, and physicians' knowledge, attitudes, and practices related to syringe access, overdose reversal, and other harm-reduction interventions associated with injection drug use. The data were double entered for accuracy and analyzed using SAS software. All protocols were reviewed by The Miriam Hospital and Temple University Institutional Review Boards (IRBs).

### Analysis

Odds ratios (ORs) are presented to describe demographic differences between responses and non-responses. We also described overall frequencies of responses on survey questions. We then focused on the outcome measure, which was a positive response to the question, "*Would you consider prescribing syringes/needles to an IDU as part of an effort to prevent the spread of blood-borne disease?*" We conducted bivariate comparisons of physicians who were willing and those who were not, by other variables, using odds ratios and 95% confidence intervals. Multivariate logistic regression was used to determine which variables were most associated with willingness to prescribe, and were reported as adjusted OR's.

The theory of planned behavior (TPB) is a socio-psychological model that has been successfully employed to assess and predict physician behavior. [17-19] The TPB posits that the intention to engage in a given behavior is a strong predictor of actual behavior, and that intention can be measured as a function of three characteristics of the actor: attitudes towards the behavior, beliefs about the ability to successfully perform the behavior and achieve the desired outcome (perceived control), and perception of peer attitudes towards the behavior (subjective norms). [20] In our analysis, the model holds that physicians' willingness to prescribe syringes to drug-using patients is a function of 1) their attitudes toward syringe prescription; 2) whether it would be feasible and useful for them to prescribe syringes (perceived control); and 3) how their colleagues and patients would react to the behavior (subjective norms). To perform the TPB analysis, eleven of the Likert-scaled agreement items were assigned by investigator consensus to one of three categories: 1) Attitudes, 2) Perceived Control, or 3) Subjective Norms. Questions included in each category are listed in Table 1. Analysis included using alpha-reliability coefficients for scale development and summary. Individual items were rescaled to 0–100 and then averaged to create final composite scales. Logistic regression models were used to evaluate the magnitude of associations between willingness to prescribe and TPB domains.

**Table 1:** Variables included in Theory of Planned Behavior Model

Attitudes:

- Prescribing sterile syringes is an important tool in stopping the spread of blood-borne disease among IDUs - Malpractice insurance should be higher for MDs who prescribe syringes - It is right to disobey the law for a compelling reason such as preventing an IDU from getting or transmitting HIV

Perceived Control

- No one would ever find out if I prescribed syringes to my IDU patients - IDUs will use sterile needles if they are made readily available - There is a legitimate medical reason for IDUs to obtain sterile syringes - I help the community by prescribing syringes to IDUs

Subjective Norms

- I would feel embarrassed justifying prescribing syringes - My colleagues believe you should obey law even if you disagree - People whose opinions I respect would approve of my bending the rules to prevent HIV among my drug-using patients - Colleagues whose judgment matters to me would disapprove of my providing syringes to IDU

## Results

Our study sample included a 1.5% (3435) geographically representative sample of AMA master file physicians from four major groups: Primary Care, Pediatrics, Emergency Medicine, and Specialists. One hundred forty-six (4.3%) individuals from our sample were either deceased or retired and 442 (12.8%) were unreachable because of incorrect contact information. This left 2847 eligible physicians for our study. Two hundred eighty-eight physicians (10.1%) refused to participate, while 69.2% (n = 1971) were non-responders. Thus, a total of 588 (20.6% of eligible) physicians responded to the survey. Two hundred sixty-six physicians (9.3%) responded after the 1^st ^mailing. Telephone numbers were available for 1636 physicians, (57%) of whom were faxed the questionnaire; 199 (7.0%) responded. An additional 4.3% responded after the 2^nd ^(n = 64) and 3^rd ^(n = 59) mailing.

Demographic characteristics of the responders compared to the non-responders can be found in Table 2. Responders were more likely to be white, female, younger, and less likely to be a primary care physician. No significant differences were found between responders and non-responders regarding region of residence in the United States or the nature of laws pertaining to syringe acquisition in their state.

**Table 2:** Demographic data comparing respondents to non-respondents (N = 3435)

	Respondents (N = 588)	Non-Respondents (N = 1971)	Chi-square p-value
Gender			
Female	29.8% (175)	24.9% (491)	0.01
			
Age (mean ± std)			
Age:	49.3 ± 11.2	51.1 ± 12.4	0.0009
			
Specialty			
Primary	81.3% (478)	85.6% (1687)	0.0001
Emergency Medicine	12.9% (76)	8.3% (164)	
Specialist	5.8% (34)	6.1% (120)	
			
Region			
Northeast	26.2% (152)	24.7% (487)	0.75
South	26.7% (155)	28.8% (568)	
Midwest	23.2% (135)	23.1% (455)	
West	23.9% (139)	23.4% (461)	
			
Homestate syringe law			
Strict Prohibition	27.0% (149)	30.4% (599)	0.12
Access Intermediate	40.1% (221)	40.6% (800)	
Open access syringe sales	32.8% (181)	29.0% (572)	
			
Syringe Exchange Program			
Legal SEP	52.8% (309)	50.0% (986)	0.12
SEP w/o claim to legal	27.2% (159)	26.1% (514)	
No SEP operating	20.0% (117)	23.9% (471)	

Responses to questions related to IDUs in physicians' practices, as well as physicians' knowledge, attitudes, and practices related to drug users are found in Table 3. Forty percent reported having IDUs in their practice, but only 28% reported that they always raise the issue of drug use when reviewing a patient's medical history. Forty-seven percent reported that they would consider prescribing syringes to drug-injecting patients, though only 6 physicians reported they had actually done so without any other indication of medical need other than injection drug use, such as diabetes. Few (7.4%) reported knowing of other physicians who prescribed syringes. Most (79%) respondents did not report knowing whether prescribing syringes was legal in their state. Over 90% attributed the sharing of syringes among IDUs to barriers to access, but most also responded that the behavior was attributable to the influence of "the drug culture" and lack of information. When asked about syringe availability in their home state, either through needle exchange programs (NEP), or over the counter (OTC) pharmacy sales, the majority (>50% across all categories of respondents) reported "don't know" (data not shown).

**Table 3:** Univariate responses to survey questions

	Total	Yes		No		Don't know	
	n	n	(%)	n	(%)	n	(%)
Do you have injection drug users (IDUs) as patients in your practice?	571	232	40.6%	339	59.4%	-	-
Is it legal in your state to prescribe syringes?	572	40	70.0%	82	14.3%	450	78.7%
Would you consider prescribing syringes as part of an effort to prevent blood-borne pathogens?	568	267	47.0%	160	28.2%	141	24.8%
Do you know other MDs who prescribe syringes?	571	42	7.40%	529	92.6%	-	-
							
Why do you think IDUs share needles?							
They don't know any better	509	278	54.6%	231	45.4%		
It is part of the drug culture	531	441	83.1%	90	16.9%		
They don't have access to enough syringes	537	505	94.0%	32	6.0%		
Lack of money	530	478	90.2%	52	9.8%		
Fear of going to a needle exchange facility	503	395	78.5%	108	21.5%		
							
Do you raise the issue of drug use when reviewing a patient's medical status or history?		417	98.1%	8	1.9%		
							
Do you ask your patients about their use of individual illegal drugs?		554	96.3%	21	3.7%		
							
Have you ever prescribed syringes to diabetic patients that you knew or suspected would use them to inject illegal drugs		57	10.1%	509	98.9%		
							
Have you ever prescribed syringes to patient with no other indication that you suspected would use them to inject illegal drugs to prevent spread of disease?		19	3.4%	546	96.6%		

Physicians willing to prescribe syringes were similar to others in gender, age, year of graduation, and specialty. Geographical differences were more pronounced, however, with those practicing in the South and Midwest being less likely to consider prescribing than those working in the West and Northeast (Table 4). Physicians who reported having IDUs in their practice were 1.5 times (95% CI: 1.02–2.29) more likely to consider prescribing syringes and 2.9 times (95% CI: 1.13–7.39) more likely if they asked their patients about syringe sharing. The strongest predictor of being willing to prescribe was the physician's belief that IDUs share because they do not have access to sterile syringes, both in the univariate and multivariate model (OR 4.6; 95% CI 2.0–10.9 and AOR 4.75; 95% CI 2.0–11.4 respectively). Physicians who reported knowing how/where syringes were legally available to IDUs in their area were significantly more likely to be willing to prescribe syringes than those who did not know or who believed there was no legal access. This held true both in univariate and multivariate analyses. Willingness to prescribe syringes was significantly associated with having prescribed syringes to a person with diabetes who might be an IDU (OR, 2.8; 95% CI, 1.3–6.2) and knowing someone who prescribes syringes to IDUs (OR, 3.8; 95% CI, 1.5–10.1); although in the final model, prescribing syringes to an IDU person with diabetes was marginally significant (i.e., included 1.0 in the confidence interval (AOR, 2.3; 95% CI, 1.0–5.2)).

**Table 4:** Bivariate and multivariate analyses comparing physicians willing to prescribe syringes by other variables

Variable (n)	willing to prescribe (%)	(n)	OR	95% CI	Adjusted OR	95% CI
Gender						
Female	64%	125	1.09	0.7–1.7		
Male	62%	302				
Specialty						
Not Primary (85)	64%	54	1.1	0.7–1.7		
Primary (342)	62%	213				
Region						
Northeast (115)	70%	81	0.9	0.5–1.6		
South (110)	54%	59	0.4*	0.2–0.8		
Midwest (102)	54%	55	0.4*	0.2–0.8		
West (95)	73%	69				
Syringe status						
Strict prohibition (103)	69%	71	1.65	1.0–2.8		
Open access syringe sales (142)	65%	92	1.37	0.9–2.2		
Intermediate Access (157)	57%	89				
SEP status						
Legal Sep (230)	67%	154	1.6	0.9–2.7		
SEP w/o claim to legal (116)	59%	68	1.1	0.6–2.0		
No SEP operating (79)	56%	44				
IDUs in practice						
Yes (181)	68%	123	1.5*	1.0–2.3		
No (239)	58%	137				
Ask IDUs about sharing needles						
Yes (168)	70%	118	2.9*	1.1–7.4		
No (20)	45%	9				
IDUs share because they don't have sterile syringes						
Yes (373)	65%	242	4.6*	2.0–10.9	4.8*	2.0–11.4
No (26)	45%	12				
IDUs share because they do not have money						
Yes (352)	65%	229	2.3*	1.2–4.3		
No (44)	45%	20				
Awareness of access syringe exchange in location of practice						
Yes (104)	71%	74	1.6	1.0–2.6		
No (91)	63%	57				
Don't know (226)	58%	131				
Awareness of over-the-counter pharmacy syringe sales						
Yes (72)	74%	53	1.8	1.0–3.2		
No (100)	65%	65				
Don't know (247)	59%	146				
Awareness of syringe prescription by physician						
Yes (115)	72%	83	1.8*	1.1–2.9	1.8*	1.1–2.9
No (94)	59%	55				
Don't know (211)	59%	124				
Syringe prescription is legal						
Yes (36)	83%	30	1.3	0.8–2.1		
No (390)	61%	238				
Ever prescribed to IDU with Diabetes						
Yes (42)	81%	34	2.8*	1.3–6.2	2.3	1.0–5.2
No (380)	60%	228				
Ever prescribe to IDU w/o indication						
Yes (15)	80%	12	2.5	0.7–9.1		
No (406)	60%	244				
Know another MD who prescribes to IDU						
Yes (34)	85%	29	3.8*	1.5–10.1		
No (390)	60%	234				

*Significant based on 95% CIs

Table 5 presents the results from TPB analyses. Willingness to prescribe syringes was significantly associated with each of the theory of planned behavior domains, with perceived control being the strongest predictor, both alone (OR = 2.9; CI: 2.4–3.7) and in the final model (OR = 2.0; CI: 2.0–2.5). Attitudes towards care for IDUs and perceptions of peer attitudes were also robust predictors of physician willingness to participate in syringe prescription programs. Physicians who regarded prescribing as an important tool for preventing HIV, and those who felt strongly enough to approve of "civil disobedience" if the practice were illegal, were more likely to be willing to consider prescribing than those who did not. Conversely, those who would be embarrassed about prescribing, and who felt their peers would disapprove of prescribing or breaking the law, were less likely to be willing to participate in such programs.

**Table 5:** Theory of Planned Behavior Predictors (n = 427)

	MDs willing Rx (n = 267)		MDs not willing (n = 160)		unadj OR	95% CI	adj OR*	95% CI
Measures	n	%	n	%			(n = 425 for model)	
Attitude Scale (3 item)					2.45	2.05–2.94	1.81	1.47–2.22
> = 50	200	75.2%	33	20.6%				(chisq = 31.9)
<50	66	24.8%	127	79.4%				
					2.24	1.87–2.70	1.53	1.23 – 1.89
Subjective Norms Scale (4 item)								
= 50	203	76.6%	56	35.0%				(chisq = 14.7)
<50	62	23.4%	104	65.0%				
					2.93	2.36–3.65	2.00	1.59 – 2.51
Perceived Control Scale (4 item)								
> = 50	206	77.7%	33	20.6%				(chisq = 35.4)
<50	59	22.3%	127	79.4%				

*odds ratio and 95% CI are for a 10 point increase in the scale

ROC for model = .902

## Discussion

Drug users in need of drug use-related care regularly seek primary care services and specialty care throughout the nation. For IDUs who are not currently willing to enter addiction treatment, two of the most pressing medical needs are access to sterile injection equipment and assistance in adopting sterile injection practices. Their regular health care providers can and should take steps to encourage treatment and help them minimize the harms of ongoing injection drug use.

This is the first national survey of physicians' attitudes and behaviors related to prescribing syringes to IDU patients to prevent the spread of disease. Our data are similar to prior findings that physicians, despite never actually having prescribed syringes to IDUs, would consider doing so. [13-15] This study highlights that physician syringe prescription is an acceptable practice for many practitioners sampled in this nationwide survey. More importantly, the findings tie syringe prescription to a range of good practices and favorable attitudes in the treatment of addiction in the course of regular medical care: willingness to prescribe syringes was strongly associated with speaking to patients about drug use and about syringe sharing, and being aware of the problem of syringe access in the area.

The Theory of Planned Behavior model that we used to identify factors influencing physician prescription of syringes suggests a number of steps that would increase physician willingness to prescribe syringes. Providing physicians with the information that prescribing syringes can benefit patients and community health might impact attitudes and greater perceived behavioral control of physicians in adopting syringe prescription practices. The law governing syringe possession and prescription was a factor in perceived behavioral control and subjective norms. Physicians will be more likely to prescribe syringes if they know that doing so is legal. Steps by public health agencies, professional bodies, and regulatory authorities to educate physicians that prescribing syringes is a legitimate medical practice [1,11] would promote providers' willingness to participate in syringe prescription programs. Moreover, given that our data showed physician willingness to prescribe was influenced by knowledge of their patients' drug using behavior, interventions directed toward standardizing physician knowledge of drug use practices may also improve their willingness to prescribe syringes.

The primary limitation of this study is the low response rate of 20.6% in our sample despite multiple, intensive inquiries. National physician surveys whose methodology we adopted and implemented in this study reported response rates between 44–65%, [21-23] however, physician surveys on topics related to substance abuse and injection drug use in particular have tended to have lower response rates. [24-26] Unfortunately, based on the low response rate, results are not generalizable to physicians in general, or to physicians who work in the subspecialties we sampled from. What is compelling about the data is that among physicians that may have a commitment to substance using patients that *did *respond, this study reveals a fundamental lack of knowledge about the culture of IDUs and their behaviors, as well as state syringe prescription laws.

How physicians, individually and as a profession, regard drug users is also an important factor in their willingness to prescribe. The statements of major medical associations in support of prescribing syringes are of immediate importance in these domains. More generally, primary and continuing medical education across all specialties is a key venue for addressing the stigma of drug use and its negative impact on patient and community health. To influence physicians earlier in the pipeline, providing substance use and addiction research as part of medical school education could formally present addiction as a clinical condition as opposed to having their first encounter with drug use be during an Emergency Department clinical rotation. Through a better understanding of the physiology of addiction, providers can be better equipped to define treatment goals and outcome measures, which include options other than simple abstinence. Given that IDUs tend to seek health care in later stages of adverse health outcomes and are more likely to receive care in the emergency room as opposed to a clinic, targeted training for certain physicians (e.g., emergency department physicians) based on their potential interaction with IDUs may increase the likely impact on drug using populations.

## Conclusion

Physicians within their medical practice have an important opportunity to intervene in substance abuse behavior on an individual level. Prescribing syringes to IDUs should figure among such interventions because it can help prevent the transmission of bloodborne disease and enhance overall IDU care. The physicians in our study were generally amenable to participating in syringe prescription programs, but physician willingness to act can be supported by better communication of what constitutes evidence-based practice, alleviation of legal concerns, and explicit validation by peers and professional organizations. Requiring substance abuse as a subject in medical training and continuing medical education would also promote better care for IDUs. Interventions that seek to address physician attitudes toward IDUs and provide state-of-the-art knowledge on the physiology of addiction could be the first step to garnering attention and renewed focus to the need to optimize IDU health care. Other strategies to improve physician knowledge include mandatory substance abuse training in medical school and continuing medical education programs which promote better care for IDUs.

The success of the IDU care programs established to date demonstrates that even a few committed physicians can make a big difference, but IDU care will remain sub-optimal–and public health will suffer–unless more physicians understand and implement harm-reduction intervention strategies like syringe prescription.

## Competing interests

The authors declare that they have no competing interests.

## Authors' contributions

GM has made the following substantial contributions: conception and design, analysis and interpretation of data, drafting of the manuscript, obtaining funding, supervision.

DD: analysis and interpretation of data, critical revision of the manuscript for important intellectual content, administrative, technical, or material support.

JR: conception and design, analysis and interpretation of data, critical revision of the manuscript for important intellectual content, obtaining funding.

CB: acquisition of data, critical revision of the manuscript for important intellectual content, administrative, technical, or material support, supervision.

LT: conception and design, analysis and interpretation of data, critical revision of the manuscript for important intellectual content, obtaining funding.

LB: analysis and interpretation of data, critical revision of the manuscript for important intellectual content.

SB: conception and design, analysis and interpretation of data, critical revision of the manuscript for important intellectual content, obtaining funding.

This work was supported by a grant from the Substance Abuse Policy Research Program of The Robert Wood Johnson Foundation. The views expressed are those of the author(s) and do not imply endorsement by the Substance Abuse Policy Research Program or The Robert Wood Johnson Foundation.
